# Plasma Exchange for Treatment of Refractory Demyelination

**DOI:** 10.15844/pedneurbriefs-34-16

**Published:** 2020-12-09

**Authors:** Michael Cronin, Mark S. Wainwright

**Affiliations:** 1 Pediatric Neurocritical Care and Division of Pediatric Neurology, Department of Neurology, University of Washington, Seattle, WA

**Keywords:** Plasma Exchange, Neuroinflammation, Demyelination

## Abstract

Researchers from the National Pediatric Hospital in Buenos Aires, Argentina, describe their experience with therapeutic plasma exchange (TPE) for refractory inflammatory central nervous system (CNS) attacks in children over the course of the last 15 years.

Researchers from the National Pediatric Hospital in Buenos Aires, Argentina, describe their experience with therapeutic plasma exchange (TPE) for refractory inflammatory central nervous system (CNS) attacks in children over the course of the last 15 years. The authors conclude that TPE is an effective treatment for severe CNS inflammatory demyelinating events that are not responsive to steroids and that the overall frequency of adverse events associated with therapy is low.

The authors analyzed a total of 78 children with confirmed acute CNS episodes who were treated with TPE. They assessed improvement after TPE using a series of standardized functional assessments across four domains. These assessments were conducted before, immediately following, and then 3 and 6 months after TPE. They found that 72% of patients (56 of 78) had moderate to marked improvement at the end of their first treatment with TPE and 82% of patients (56 of 68) after three months of treatment. The authors report four adverse events that they characterize as "serious" and 27 which they classify as "mild to moderate" out of 65 patients who underwent a total of 524 procedures. [1]

COMMENTARY. Treatment of refractory CNS inflammatory disorders in children is variable, and data supporting these practices is lacking. This study is an essential contribution to the pediatric literature on inflammatory CNS attacks, although there are some limitations as a retrospective analysis. First, there is no control group, and children who suffer acute demyelinating attacks can often recover independently of the treatment they receive, even those who present with profound impairment [2]. When describing the persistence of benefit at six months, the authors report that a higher percentage of patients experienced recovery (82%) in the TPE group. However, the absolute number of patients (56) remained the same because the patients who did not experience improvement did not continue to receive TPE therapy and were excluded from subsequent analysis. This raises the question of whether clinical improvement in the TPE group can be attributed to the TPE therapy itself.

TPE carries a risk for complications. Several of the events the authors classified as "mild" (hypotension, catheter-related infection, symptomatic hypocalcemia) are also potentially life-threatening. Complications from the placement of central venous catheters in critically-ill children occur at rates that can be up to as high as 22% [3]. Younger children are also more likely to experience complications, including accidental removal of the catheter itself [3]. Younger children also require sedation to place a central venous catheter, further placing them at risk for additional complications. Finally, TPE's successful course requires expertise in many areas that may not be available at all centers [4].

Data from another large series suggest TPE should be used with caution in demyelinating disorders. In a retrospective study of 283 Canadian children with acute demyelinating attacks, most (< 90%) recovered, and only four received TPE [2]. In the series by Savransky et al., one patient died due to pulmonary embolism, thought to be attributable to central line placement [1]. The use of baseline and repeat assessments are strengths of this study and lend weight to the author's conclusion that TPE is an effective treatment for this disorder, even when delayed up to six months after symptom onset. The attention to the potential for TPE complications is a reminder that in seeking to help children with these debilitating illnesses, we must recognize potential risks in addition to the uncertain benefits.

## Disclosures

The authors have declared that no competing interests exist.

## References

[1] Savransky A, Rubstein A, Rios MH, Vergel SL, Velasquez MC, Sierra SP (2019). Prognostic indicators of improvement with therapeutic plasma exchange in pediatric demyelination. Neurology.

[2] O'Mahony J, Marrie RA, Laporte A, Yeh EA, Bar-Or A, Phan C (2015). Recovery from central nervous system acute demyelination in children. Pediatrics.

[3] Karapinar B, Cura A (2007). Complications of central venous catheterization in critically ill children. Pediatr Int.

[4] Misanovic V, Pokrajac D, Zubcevic S, Hadzimuratovic A, Rahmanovic S, Dizdar S (2016). Plasmapheresis in Pediatric Intensive Care Unit. Med Arh.

